# Deaf Mute Restored to Hearing by an Attack of Variola

**Published:** 1863

**Authors:** J. J. Samuels

**Affiliations:** Marion, Ill.


ARTICLE XI.

### DEAF MUTE RESTORED TO HEARING BY AN ATTACK OF VARIOLA.

MARION, ILL., *April 16th*, 1863.

PROF. N. S. DAVIS,

DEAR SIR:—The following case of variola, which occurred recently in my practice, has connected with it a circumstance which may be of some interest to the profession, although, the course and termination of the disease itself presents nothing unusual:—

George H. Dickinson, a deaf mute, aged 45 years, was seized with the usual symptoms of an attack of small-pox, 14th March last. There was nothing remarkable either in the eruptive fever, the appearance of the eruption, (which was confluent,) the maturation of the pustules or secondary fever. The patient passed on to convalescence, and is now completely recovered. But, what is rather strange, the patient, during his illness, became able to hear with the left ear, the other remaining closed, and could talk almost immediately after he could hear. While visiting him on the evening of the 21st, (which was the fourth day of the eruption,) he wrote upon his slate these words: "I heard to-day for about twenty minutes as plainly as you ever did, how do you account for it, doctor?" I paid but little attention to this, and hastily replied, that I supposed the inflammation in the throat had affected the ear through the eustachian tube.

On the evening of the 22d, he complained of pain in the left ear; and, on examination, I discovered a slight discharge of muco-purulent matter from the external meatus. One of the attendants informed me that the patient heard the noise made by the lowing of a cow near his room, and had also heard thunder in a storm-cloud that passed over.

On the morning of the 23d, while at his bedside, I observed his attention was attracted by the singing of birds near his room. I told him that it was the birds singing, and asked him what kind of music they made. In reply, he whistled in imitation of them. This was the first satisfactory evidence I had had that he really could hear. He still complained of pain in his left ear. Being called to the country, I did not see him on the 24th, he being visited by my preceptor and partner, Dr. A. N. Lodge. On entering the door of his room on the morning of the 25th, I bowed and inquired how he got along, to which he replied "bully;" and it took him but a few minutes to tell me the following, which I had already learned from Dr. Lodge:— "When he awoke on the morning of the 24th, his head was filled with very loud and confused sounds. The confusion and astonishment, for the moment, produced a distraction of the mind. On regaining himself, he wished some explanation of the phenomena, he called out distinctly 'where is doc.' He also discovered that he could hear his attendants talk, and could repeat the words after them." It took him but a few hours to learn a sufficient number of words to converse on ordinary subjects; indeed, it appears to me that he could speak words that he never heard spoken. He now hears and speaks with as little difficulty as any one.

The following questions have occurred to me concerning the case:—1st. Did he never hear before? 2d. What influence had the disease on the organs of hearing? 3d. How could he so readily speak after he could hear? As regards the first question. I found the evidence that he had not heard or spoken for 25 years, conclusive. Many of our best citizens have know him for that period without a suspicion ever arising in their minds that he could hear or speak. Further back than that, we have only his evidence, which, in his own language is: "So far as my recollection of myself reaches back, I never did hear; and, to the best of my knowledge, my parents taught me that I never could hear." So we are left to suppose either that he could hear in infancy, but that by disease it was destroyed while very young, or that he never could hear. I leave each one to their own supposition as to what influence the disease exerted on the function of hearing. I could not now, and, perhaps, never, give a better answer than I gave to the patient, when he requested an explanation.

I hope men more competent than myself will endeavor to explain this matter, in regard to the third question,—"how could he so readily speak after he could hear." I might state that Dickinson has a fine education, and completely mastered, among other things, that most difficult science, labiaology, which was taught him by the late Dr. J. K. MITCHELL, of Philadelphia, under whose instructions he was for four years. A brother and a sister of Dickinson's were also deaf mutes; his parents were cousins. He has been married once, his wife was a deaf mute also. He has two living children, a son and a daughter, the former aged five and the latter four years, and both are blessed with perfect hearing and speech.

J. J. SAMUELS.

ARTICLE XII.

### A DEATH FROM CHLOROFORM.

Reported by E. ANDREWS, M.D., Professor of Surgery in Chicago Medical College.

Deaths from the administration of Chloroform are so seldom fully reported, that every effort needs to be made to fully elucidate the phenomena and rationale of the accident, in order that the obscurity which still shrouds the subject may be dissipated as soon as possible.

In the following case, although I did not witness the death, I was personally acquainted with the attendants, whom I carefully questioned respecting circumstances, and I had the pleasure of making the *post mortem* examination with my own hand.

Mr. D—— was about sixty years of age, and of intemperate habits, but not known to be otherwise diseased. After an unusually prolonged debauch he applied to a physician to shield him from the impending horrors of *delirium tremens.* He was found to be rational, with no delusions of the senses, but with a feeble and frequent pulse, and a well-marked trembling of the muscles of voluntary motion.

The physician prescribed a mixture of tinct. cinchona, laudanum, and alcohol, to be frequently repeated in small doses.

He continued two days, under this treatment, with no material change, but on the third day he became wild with the usual form of *mania a potu,* and refused, absolutely and determinately, all medication. Some fourteen hours or more were wasted in this manner, during which he took neither medicine nor food. At this juncture, my advice was sought, and I endeavored to induce the patient to yield to strong persuasion. Failing entirely in this, I advised that he should be placed under the influence of chloroform, sufficiently to overcome his obstinacy, and that then suitable tonics, together with a powerful opiate, should be administered. I judged that unless his excitement should be subdued speedily, there was no hope of any recovery, as he had already almost exhausted his vital energies; and it seemed to me that the tranquilizing effect of anæsthesia might serve to give time for a strong anodyne to commence its influence.

I then left, and in accordance with my advice the anæsthesia was attempted by the physician and attendants. The administration was begun cautiously, admitting an abundance of air with the vapor, but the patient made such a violent and continued resistence that it was found almost impossible to continue it. After much difficulty, however, he was partially secured, and the chloroform was given very rapidly and copiously, in order to subdue his resistence. In a short time he became quiet, when the medicine was placed in his mouth. This re-arroused his consciousness, and he spit it violently out, and re-commenced his struggles. The chloroform was then copiously re-applied until he became quiet. At this juncture the medicine was given and swallowed in an easy and natural manner, and the patient lay upon the bed respiring quietly and freely. In about three minutes he was noticed to be pale and ghastly in the countenance, but still respiring. The paleness momentarily increased, and in five minutes more both respiration and the pulse ceased, and the beating of the heart could not be felt in the chest. There was no evidence of asphyxia, but the physician in attendance thought it his duty to make use of artificial respiration until all hope of resuscitation had departed. His efforts, however, were unavailing, as not the slightest sign of returning vitality presented itself.

The chloroform, in this case, was from a good article, which had been given, without evil effects, to several other patients. It was administered upon a cloth folded into a cup-form, and held over the face. The administration, owing to the difficulty of restraining the patient, was petty freely pressed.

The *post mortem* examination revealed no physical signs to account for the accident. The heart was found nearly empty in all its cavities. Long white clots were, however, attached to its valves in an unusual manner, and may, possibly, have interfered with their action. On the right side, the clot had its thickest part in the auricle, and its attachment to the *chordæ tendinæ* of the tricusped valves beneath. Instead, however, of streaming off like a banner in the normal direction of the blood-current, *i. e.*, through the pulmonary artery, it presented two trains, one of which extended through the auricle and thence down into the ascending vena cava; and the other took the usual course up the pulmonary artery. As these banner-like white clots cannot extend themselves in a direction contrary to the current of the blood, I can only account for their double direction by supposing, that the formation of the first portions entangled and tied open the tricuspid valves, so that the blood was no longer regularly pumped through, but simply churned in and out of the ventricle, most of it returning at each contraction to the cava, and only a little passing up the pulmonary artery. The fact that the train of the clot which extended towards the vena cava was the largest of the two, gives countenance to this supposition. This also would account for the fact, that the lungs and the left side of the heart were found almost empty of blood. There was a small white clot found attached to the mitral valves, and streaming up into the aorta in the usual manner.

The lungs were dark with the carbonaceous stains of old age, and presented evidences of old pleuritis, but were nearly destitute of blood, and healthy in their structure. Owing to the objections of friends, the brain could not be examined. The liver and other abdominal viscera presented no unusual appearances.

It is obvious, both from the symptoms before death, and from the phemomena presented at the autopsy, that the death, in this case, took place by syncope, and not by asphyxia, as is usually supposed to be the mode of fatal chloroformization. If the formation of the clot which enlarged the valves of the right ventricle was commenced before the patient was in *articulo mortis*, it would account for the failure of the heart to propel the blood and for the consequent syncope, of which, the patient died. If, however, as many suppose, these white clots only form in the presence of immediate death which is impending from other causes, then, the explanation is as obscure as ever. I have observed that chloroformized animals sometimes die of syncope in a similar manner, but I did not observe in them any peculiar clots.

It is a settled fact, that chloroform is a more dangerous agent of anesthesia than sulphuric ether, but yet the danger is not great. About a thousand cases of its administration fell under my notice while I was in the Army, no one of which resulted fatally. I have been able to hear of only one fatal case in the Western Armies since the war commenced. On the whole, although the exigencies of the battle field will always necessitate the use of pure chloroform in the surgery of war, I advise great caution in its use in civil practice. Probably, the best method of anesthesia is to have present both ether and chloroform. Let the administration be commenced either with ether alone, or with ether mixed with one-fourth part of chloroform. If the patient proves too insusceptible to this agent, and cannot be reduced to anesthesia by it alone, more chloroform can then be added, or even the pure chloroform be administered until the desired effect is obtained.

